# Effects of manual hyperinflation with rib cage compression and endotracheal suctioning on arterial blood gas parameters in mechanically ventilated patients at a university hospital, Egypt

**DOI:** 10.1186/s43168-021-00096-5

**Published:** 2021-11-03

**Authors:** Nahla Shaaban Khalil, Manal Sayed Ismaeel, Ahmed Hassan Askar, Mohamed Soliman Sayed

**Affiliations:** 1 Critical Care & Emergency Nursing, Faculty of Nursing, Cairo University, Giza, Egypt; 2 Department of Critical Care Nursing, Faculty of Nursing, Britain University, Cairo, Egypt; 3 Critical Care Medicine Department, Cairo University, Giza, Egypt

**Keywords:** Manual hyperinflation, Rib cage compression, Endotracheal suctioning, Arterial blood gas parameters, Mechanically ventilated patients

## Abstract

**Background:**

Manual hyperinflation and expiratory rib cage compression are methods of chest physiotherapy. They are commonly applied but their value and their early utilization managing mechanically ventilated remain questionable. The purpose of the study was to investigate the effects of manual hyperinflation with rib cage compression and endotracheal suctioning on arterial blood gas parameters in mechanically ventilated patients.

**Results:**

Seventy mechanically ventilated patients at a teaching hospital in Egypt were assigned to the clinical trial study. The age of more than half of the studied samples ranged between 60 and 69 years with a mean age of 53.64 ± 16.44 years. Initially, the ABG parameters were assessed. Then, the patients were exposed to manual hyperinflation for 5 min and 20 min external expiratory chest compression followed by endotracheal suctioning. Later, ABG parameters were assessed again and compared to the initial one. The findings revealed significant differences in ABG parameters before and after completion of MHI and ERCC and ETT suctioning in relation to PaO_2_ (*t* = 3.892) and SaO_2_ (*t*= 5.904). Also, it showed significant improvement in PaO_2_ and SaO_2_ after the completion of interventions, while no significant differences were found in other ABG parameters. This study was registered retrospectively with an ISRCTN number 39983 on 5/6/2021.

**Conclusion:**

Applying manual hyperinflation with rib cage compression and endotracheal suctioning improved mainly the arterial oxygenation parameters in mechanically ventilated patients despite no significant changes observed in other ABG parameters.

## Background

Most critically ill patients require mechanical ventilation and specialized nursing care during their stay in the intensive care unit (ICU). Accordingly, the critical care nurse should ensure that the patients who receive mechanical ventilation have continuous monitoring for oxygenation and adequate measurements of arterial blood gases. Airway blockage due to the accumulation of secretions can impair gas exchange and hence decrease the partial pressure of oxygen (PaO_2_) and increase the partial pressure of carbon dioxide (PaCO_2_) in the arterial blood [1].

Gas exchange impairment may cause serious complications such as respiratory acidosis, cyanosis, and lethal cardiac dysrhythmias, this problem is more severe in patients connected with mechanical ventilation, because the intubation causes irritation of the mucus-producing goblet cells, causing increased of mucous [2]. Therefore, prevention of such complications is among the goals of care for these patients. To achieve this goal, providing sufficient humidity, proper suctioning, chest physiotherapy, changing position frequently, early ambulation, and adequate hydration are some of the required nursing interventions [3].

Manual hyperinflation (MH) is a form of positive pressure ventilation using a rebreathing bag. It is used primarily to stimulate cough in patients with either poor or absent cough reflex or when there is atelectasis or excessive bronchial secretions. The aim of utilizing hyperinflation is to re-expand the collapsed alveoli, mobilize and eliminate the overflow of bronchial secretions, and make the oxygenation better in mechanically ventilated patients by making larger volume breaths [4, 5].

Expiratory rib cage compression is one method of chest physiotherapy which involves chest squeezing by hands during expiration and releasing that at its end to help in the mobilization of lung secretions, to make active and more comfort inspiration, and to improve alveolar ventilation. This technique increases forced expiratory volume by 30% and leads to the resting of the expiratory muscles. Most of all, the technique is quite safe, as it has been employed in some patients for more than 3 years with no complications [3]. Therefore, this technique can be used before the patients’ endotracheal suctioning interventions, and it is widely used with mechanically ventilated patients to prevent and/or to treat atelectasis [3]. In addition, removing secretion is essential because accumulated secretions intervene in gas exchange and may delay recovery; coughing can be initiated voluntarily or by reflex [6]. After performing manual hyperinflation and expiratory rib cage compression, arterial blood gases are carried out to assess the patient’s acid-base balance and oxygenation. Arterial blood is utilized because it gives a more accurate reflection of gas exchange in the lung than in the venous blood [7]..

Through our clinical practice and experience in the university hospital, it has been observed that mechanically ventilated patients develop dangerous and repeated complications that cause delayed recovery and prolongation of hospital stay length. As a result, there are various techniques of respiratory therapy utilized in critical care units to minimize these complications. One such technique is manual hyperinflation (MH), which is advised to optimize alveolar ventilation by making gas exchange more improved through liquefying and moving the pulmonary secretion making endotracheal suctioning more comfortable and improve lung compliance. Furthermore, this data could be beneficial in maintaining a cost-effective patient care especially in such critical care units as it might shorten hospital stay and safeguard patients against any life-threatening complications. Also, it provides database that can be utilized by health care professionals in the provision of future care for such a group of patients, and it is hoped that this effort will generate attention and motivation for further investigations into this area.

### The aim of the study

The aim of this study was to examine the effects of the intervention of manual hyperinflation with rib cage compression followed by endotracheal suctioning on arterial blood gases in patients receiving mechanical ventilation.

## Methods

Pre/post-quasi-experimental design was utilized in this clinical trial study. Quasi-experimental design is a type of research design in which the treatment and control or comparison groups are not created using random assignment procedures. It involves the manipulation of an independent variable and the specification of a test hypothesis [8].

To fulfill the aim of the study, the following research hypothesis is formulated: Mechanically ventilated patients who expose to the intervention of manual hyperinflation with expiratory rib cage compression followed by open endotracheal suctioning will have improved arterial blood gas parameters than before the intervention.

A prospective study was conducted utilizing a purposive sample of 73 adult male and female mechanically ventilated patients who spent for more than 24 h in different medical critical care units were enrolled in the study. The duration of the study was 5 months. The exclusion criteria included length of ICU stay ≤ 24 h; positive end-expiratory pressure (PEEP) > 8 cmH_2_O, pneumothorax, or a history of pneumothorax; chest tube drainage; chest trauma; brain edema, raised intracranial pressure, or the potential to develop pathologically raised intracranial pressure; unstable cardiovascular status (defined as systolic blood pressure < 100 mmHg or > 180 mmHg, mean arterial pressure < 70 mmHg or > 110 mmHg, or HR < 70 bpm or > 120 bpm); and spinal cord injury.

### Settings

The study was carried out in three medical intensive care units at Al Manial University Hospital in Egypt during the years 2018–2019.

### Tools of data collection

Two tools were utilized for data collection. These tools were designed by the investigator and revised by a panel of three experts in critical care and emergency nursing; these tools are as follows:

Tool 1: patient’s demographic and medical characteristics data which included the patient’s age, gender, date of admission, past medical history, medical diagnosis, modes, and parameters of mechanical ventilation.

Tool 2: arterial blood gas (ABG) monitoring chart is performed to evaluate the patient’s acid-base balance and oxygenation. It was used before and after implementation of MHI, ERCC, and endotracheal suction (25min).

An official permission to conduct the study was obtained from the heads of critical care units and from the Faculty of Nursing Institutional Ethics Committee with Approval number FWA00019803. Also, written consent was obtained from the patients/their relatives after description and clarification of the nature and purpose of the study by the researchers.

A pilot study was conducted on eight adult patients who fulfill the inclusion criteria to ensure objectivity, clarity, feasibility, and reliability of the study tools. No necessary modifications were needed. Thus, the 8 patients were included in the current study. Data of the current study was collected over a period of 5 months starting from July to December 2018.

*Procedural steps of interventions*

### The procedure of hyperinflation

The steps of the implementation of MHI are as follows: Assess the patients’ vital signs to ensure they are stable and in order to detect changes in the patients’ condition, Prepare the patient by giving an explanation to minimizes any distress to the patient, thus maximizing the effectiveness of treatment, position the patient so that the lung to be treated is uppermost to optimizes ventilation to the affected lung, and assists with the drainage of secretions. Then, attach the re-breathing bag to 2 L O_2_ and make sure that the expiratory valve is working and locate a filter in the circuit between the bag and the patient and connect the manometer to prevent hypoxia. Ensure the safety of equipment to prevent cross-contamination of the bag. Set the O_2_ flow rate to15 L per minute to ensure 100% oxygen is delivered and the bag fills quickly. Put the ventilator on standby or use the pre-oxygenation suction facility to disable the alarm to prevent patient’s anxiety [9]. Then, separate the patient from the mechanical ventilator and attach the re-breathing bag to the airway through the catheter mount or to enable manual hyperinflation using two hands. At the beginning, provide a tidal volume breath and watch the patient’s chest expansion to allow the operator to obtain feeling of patients lung compliance. Make sure that sufficient tidal volume is being provided into the patients’ lungs. Next, perform MHI breaths. The manual hyperinflation breath should be maintained for at least 2 s to make sure that manual hyperinflation breaths are delivered effectively and expand the collapsed alveoli. Deflate the bag suddenly on expiration to simulate the mobilization of secretions from the periphery to the central airways. Also, repeat the procedure many times as mentioned to cause more removal of secretion and expansion of the collapsed alveoli. Explain the procedure to the patient throughout the entire intervention and always synchronize with the patient’s own ventilation to relieve stress and pain of the patient. If the patient is coughing, the expiratory pressure valve should be released to decrease the pressure developed in the lungs and minimize the risk of barotraumas. Then, perform suctioning if the patient coughs or secretions are auscultated to remove secretions preventing them being returning back into the smaller airways. Continue the above procedure until no more secretions are heard or the chest is clear on auscultation to expand the collapsed alveoli ensure clearance of secretions [9].

### The procedure of expiratory rib cage compression (ERCC)

The ERCC technique consists of manual compression of the rib cage during the expiratory phase and release from the compression at the end of the expiration, in an attempt to remove pulmonary secretions, to facilitate active inspiration, and to improve alveolar ventilation. Manual bilateral expiratory rib cage compression was performed by a single nurse, who attempted to use a consistent technique, applying the same force with each patient [10].

In the rib cage compression, the nurse performs bilateral squeezing to the lower rib cage gradually during expiration. Each rib cage compression was released at the end of expiration to facilitate comfort inspiration. Specific care was taken to make sure that compression was executed only during expiration. Therefore, this technique can be used before patients’ endotracheal suctioning interventions and it’s widely used with mechanically ventilated patients to prevent and/or to treat collapsed alveoli. In addition, removing secretion is important because it retained secretions interfere with gas exchange and may slow recovery [3].

### The open suctioning technique

This was carried out immediately after completion of manual hyperinflation, and expiratory rib cage compression requires disconnecting the patient from the ventilator, ventilator utilizing sterile aseptic technique [11].

### Withdrawing the radial arterial sample

This was carried out before and after the abovementioned interventions**.**

### Interpreting, documenting, and comparing

The blood gas parameters before and after completion of interventions.

### Statistical analysis

The patients’ data were entered into the computer and analyzed using IBM SPSS Corp. IBM SPSS Statistics for Windows, version 22.0. The statistical analysis was executed utilizing SPSS version 22. Continuous data are presented as mean and standard deviation. Categorical data are presented as number and percentage. The comparison between the two means before and after the intervention was done by paired *t*-test. All statistical tests in the present study were two-tailed with *P* considered significant if < 0.05.

## Results

As can be seen from Table 1, nearly half of the studied sample was males (53.4%), and their age ranged between 60 and 69 years with a mean ± *SD* of age 53.64 ±16.44 years. Regarding patient diagnosis and past medical history, Table 2 shows that two-thirds of patients’ diagnosis were related to respiratory problems (65.8%).

**Table 1:** Percentage distribution of the studied patients in relation to their characteristics (*N* = 73)

Item	Number	Percent
**Gender**		
Male	39	53.4
Female	34	46.6
**Age group**		
20–29	8	11
30–39	6	8.2
40–49	7	9.6
50–59	12	16.4
60–69	40	54.8

Mean ± *SD*, 53.64 ±16.44

**Table 2:** Frequency distribution of the sample according to their medical characteristics’ data (*n* = 73)

Item	Number	Percent
Medical diagnosis		
Respiratory	48	65.8
Cardiac	6	8.2
Neurology	8	11
GIT	5	6.8
Renal	4	5.5
Surgical	2	2.7
Medical history		
None	19	26
DM	3	4.1
HTN	3	4.1
IHD	6	8.2
Liver impairment	4	5.5
Renal impairment	7	9.6
CVS	1	1.4
Cancer	1	1.4
COPD	3	4

Out of 48 patients suffering from respiratory diagnosis, half of the patients suffered from a chest infection (50%), and the others distributed equally between respiratory failure (25%) and chronic obstructive pulmonary diseases (25%) (Table 3).

**Table 3:** Percentage distribution of the studied subjects who have a respiratory diagnosis (*N* = 48)

Respiratory diagnosis	Number	Percent
Chest infection	24	50
Respiratory failure	12	25
Obstructive pulmonary disease	12	25

Table 4 shows that 45.2% of mechanically ventilated patients were on AC mode of mechanical ventilation with the mean parameters as follows PEEP (5.57), FiO_2_ (51.98%), frequency (9.95), tidal volume (258.01), pressure support (6.65). Moreover, it revealed that the mean length of patients’ connection to mechanical ventilation in days before applying the intervention was 7.5 days.

**Table 4:** Frequency distribution, mean and standard deviation of modes, parameters, and length of stay of mechanical ventilation of the study sample (*N* = 73)

Variables	No. %	
	Mean	***SD***
**Ventilator modes**		
Assist control (AC)	33	45.2
Synchronized intermittent mandatory ventilation (SIMV)	11	15.1
Continuous positive airway pressure (CPAP)	29	39.7
**Ventilator parameters**		
Positive end-expiratory pressure (PEEP)	5.57	1.11
Fraction of inspired oxygen (*FiO*_2_)	51.98	10.36
Respiratory frequency (*F*)	9.95	8.1
Tidal volume (TV)	258.01	226.39
Pressure support (PS)	6.65	8.38
**Length of stay (*M*** ±***SD*)**	7.50	7.41

Table 5 reveals that there are significant differences in ABG parameters before and after completion of MHI and ERCC and ETT suctioning pertinent to PaO_2_ (*t* = 3.892) and SaO_2_ (*t*= 5.904). So, it showed improvement in PaO_2_ and SaO_2_ after completion of interventions, while no significant differences were seen in the rest of the ABG parameters.

**Table 5:** Comparison of ABG parameters before and after completion of the intervention including manual hyperinflation and expiratory rib cage followed by ETT suctioning (*n* = 73)

Intervention ABG parameters		Before MHI and ERCC and ETT suctioning	After MHI and ERCC and ETT suctioning	*T*	*P*
PH	Mean	7.39	7.39	0.201	0.841
	*SD*	0.066	0.060		
PaO_2_ (mmHg)	Mean	76.41	88.49	3.892	0.000*
	*SD*	43.37	11.35		
CO_2_ (mmHg)	Mean	40.91	40.91	1.210	0.230
	*SD*	11.12	7.22		
HCO_3_ (mEq/L)	Mean	24.20	24.14	0.147	0.984
	*SD*	5.48	5.08		
SaO_2_ (%)	Mean	96.02	98.01	5.904	0.000*
	*SD*	1.98	1.74		

*Statistically significant (if *P* < 0.05)

## Discussion

The present findings tested the research hypothesis that stated mechanically ventilated patients receiving manual hyperinflation and expiratory rib cage compression followed by endotracheal suctioning will have improved arterial blood gas parameters as compared to prior interventions. So, the present study showed that there are no significant differences in the ABG parameters regarding PH, CO_2_, and HCO_3_, while showing significant improvement in oxygenation parameters: PaO_2_ and SaO_2_. Hence, more improvement in oxygenation parameters was increased after completion of manual hyperinflation with ribcage compression and endotracheal suctioning. In harmony with our result, Elbasiouny and Raafat [12] explained that applying MHI and ERCC easily opens collateral channels within the lungs, which could theoretically recruit atelectatic lung regions and facilitate secretion mobilization, improvement in gas transfer in the lung, and improvement in the ventilation-perfusion.

On the same line, the study done by Elbasiouny and Raafat [12] investigated the arterial oxygenation response to manual hyperinflation as an added procedure to chest physiotherapy in critically ill mechanically ventilated patients is consistent with our finding that revealed significant improvement in PaO_2_ and SaO_2_ parameters after the interventions of manual hyperinflation. Similar to our result found by Kohan et al. [2] who examined the effects of expiratory rib cage compression before endotracheal suctioning on arterial blood gases in patients receiving mechanical ventilation and revealed improved levels of PaO_2_ and SaO_2_. Hence, the application of ERCC can reduce the patients’ need for oxygen and reduce the side effects of oxygen therapy [2].

In agreement with our study, a study done by Malekzadeh et al. [13] who examined the effect of lung manual hyperinflation (MHI) on oxygenation among patients following abdominal surgery and T-tube support showed that SaO_2_ and PaO_2_ was significantly higher after intervention and reported that MHI technique enhance oxygenation, ventilation, and improve lung function in the participants resulting in a decreased duration of mechanical ventilation, accelerating the process of extubation, and thus faster patient recovery.

In contrast to our result, Unoki et al. [14] who studied thirty-one intubated, mechanically ventilated patients in an intensive care unit received endotracheal suctioning with or without rib cage compression, with a minimum 3-h interval between the 2 interventions. That study found that rib cage compression prior to endotracheal suctioning does not improve airway secretion removal, oxygenation, or ventilation after endotracheal suctioning in mechanically ventilated patients. Also, our study contradicted with another study done by [15] who studied the manual hyperinflation with or without rib cage compression in mechanically ventilated patients and found no improvement in lung compliance, gas exchange, and secretion clearance in mechanically ventilated patients.

Finally, the current study findings done by [16] indicated that a simple and inexpensive manual hyperinflation using resuscitation bag with oxygen delivery and simple chest compression technique at the end of expiratory process showed significant improvement in oxygenation and sputum clearance on mechanically ventilated patients. These results, together with the observations of other trials, suggest that manual hyperinflation and expiratory rib cage compression are safe and it can help in improving arterial blood gases, weaning from the ventilator, and decreasing the time spent in intensive care units.

### Limitation of the study

We believe that a limitation of our finding is due to utilization of non-randomization and Findings are less amenable to generalization because the sample was selected from one geographical area in Egypt. Also, few Egyptian studies were done in this area of research. Therefore, further studies on a larger number of Egyptian critical care units in different hospitals may achieve significant findings.

## Conclusion

In the end, we can conclude that after applying manual hyperinflation with rib cage compression and endotracheal suctioning among mechanically ventilated patients showed significant improvement in the arterial oxygenation parameters in terms of PaO_2_ and SaO_2_% despite no significant changes observed in other ABG parameters.

## Data Availability

Not applicable

## Abbreviations

ABG: Arterial blood gases
AC: Assist control
CPAP: Continuous positive airway pressure
ERCC: External rib cage compression
ETT: Endotracheal tube
*FiO*_2_: Fraction of inspired oxygen
MH: Manual hyperinflation
PaO_2_: Partial pressure of oxygen
PaCO_2_: Partial pressure of carbon dioxide
PEEP: Positive end-expiratory pressure
SIMV: Synchronized intermittent mandatory ventilation
*F*: Frequency
TV: Tidal volume
PS: Pressure support
